# The correlations between the anchor density and the curve correction of adolescent idiopathic scoliosis surgery

**DOI:** 10.1186/s12891-019-2844-1

**Published:** 2019-10-27

**Authors:** Yu-Cheng Yeh, Chi-Chien Niu, Lih-Huei Chen, Wen-Jer Chen, Po-Liang Lai

**Affiliations:** 1 Department of Orthopedic Surgery, Chang Gung Memorial Hospital, No. 5, Fuxing St., Guishan Dist.,, 33305 Taoyuan, Taiwan; 2 Bone and Joint Research Center, Chang Gung Memorial Hospital, Taoyuan, Taiwan; 3 College of Medicine, Chang Gung University, Taoyuan, Taiwan; 4 Department of Orthopedic Surgery, Chung Shan Hospital, Taipei, Taiwan

**Keywords:** Adolescent idiopathic scoliosis, Three-dimensional curve correction, Anchor density, Posterior fusion, Pedicle screw instrumentation, Thoracic kyphosis

## Abstract

**Background:**

The optimal anchor density in adolescent idiopathic scoliosis (AIS) surgery to achieve good curve correction remains unclear. The purpose of the study is to analyze the correlations between three-dimensional curve correction and anchor density in the pedicle screw-based posterior fusion of AIS.

**Methods:**

One hundred and twenty-seven AIS patients receiving primary posterior fusion with pedicle screw instrumentation were retrospectively reviewed. Anchor density (AD) was defined as the screws number per fused spinal segment. The correlations between three-dimensional curve correction radiographic parameters and anchor density were analyzed with subgroup analysis based on different curve types, curve magnitudes, and curve flexibilities. The differences of curve correction parameters between the low-density (AD ≤1.4), middle-density (1.4 < AD ≤1.7) and high-density (AD > 1.7) groups were also calculated. Independent t-test, analysis of variance (ANOVA), and Pearson’s correlation coefficient were used for statistical analysis.

**Results:**

There were no correlations between the anchor density and the coronal curve correction or apical vertebral rotation (AVR) correction. In the sagittal plane, mild positive correlations existed between anchor density and thoracic kyphosis correction in all patients (r = 0.27, *p* = 0.002). Subgroup analysis revealed similar mild positive correlations in Lenke 1 (r = 0.31, *p* = 0.02), Lenke 1–3 (r = 0.27, *p* = 0.01), small curves (40°-60°, r = 0.38, *p* <  0.001), and flexible curves (flexibility > 40%, r = 0.34, *p* = 0.01).

There were no differences between low-density (mean 1.31), middle-density (mean 1.55), and high-density (mean 1.83) in terms of coronal or axial curve correction parameters. Low-density group has longer fused level (mean difference 2.14, *p* = 0.001) and smaller thoracic kyphosis correction (mean difference 9.25°, *p* = 0.004) than high-density group.

**Conclusion:**

In our study, the anchor density was not related to coronal or axial curve corrections. Mild positive correlations with anchor density were found in thoracic kyphosis correction, especially in patients with smaller and flexible curves. Low anchor density with longer fusion level achieves similar curve corrections with middle or high anchor density in adolescent idiopathic scoliosis surgery.

## Background

Pedicle screw-based instrumentation has gained popularity in surgery of adolescent idiopathic scoliosis (AIS) patients in the past twenty years [1–3]. From a mechanical standpoint, it provides three columns fixation of the vertebrae and shows a better curve correction for AIS patients. However, increased anchor density may lead to higher implant costs, risks of implant malposition, and blood loss [4–6].

AIS curves are complex three-dimensional deformities that require adequate correction in all three dimensions, including coronal, sagittal and axial planes. Controversies still exist in determining the relation between anchor density and curve correction in all three dimensions [7–17]. In a recent consensus, most experienced panelists agreed that less than 1.60 of the anchor density was optimal in treating AIS with smaller major curve (40°-70°) [18]. The lower safe limit of anchor density to maintain long-term adequate correction remains to be determined.

The main purpose of the present study is to determine the relations between anchor density and curve correction in coronal, sagittal and axial planes of adolescent idiopathic scoliosis surgery.

## Material and methods

### Patients

The present retrospective study analyzed a consecutive series of patients with AIS who received posterior fusion and instrumentation at our institute. This study was approved by the Institute Review Board (IRB No. 201701561B0) at our hospital. All of the patients underwent surgery between January 2009 and December 2013. The AIS patients included in this study were 1) aged from 10 to 24 years old, 2) received primary posterior fusion and instrumentation, 3) used all pedicle screw constructs, and 4) followed up for at least 48 months. The patients were excluded if any of the following criteria were met: 1) neuromuscular scoliosis, 2) infantile or juvenile idiopathic scoliosis, 3) received pedicle screws and hooks hybrid fixation, 4) received anterior fusion and instrumentation surgeries, and 5) inadequate follow-up duration.

All of the surgeries were performed by the same surgical team, comprising two attending surgeons and one rotating resident. All scoliotic curves were classified and operated according to the guidelines described by Lenke [19].

### Surgical techniques

The surgical procedures followed the technique described by Suk [20]. A standard midline approach was applied with the patient in the prone position. Intraoperative posteroanterior and lateral radiographs were used to assess the positions of K-wires as pedicle screw entry points. The pedicle screws were then inserted with the guidance of K-wires. The implant distribution was preoperatively determined based on curve magnitude, flexibility and fusion length. The preoperative planned upper instrumented vertebra (UIV), lower instrumented vertebra (LIV) would be fully inserted with pedicle screws at the bilateral pedicles. The preoperative determined reduction side (concave or convex) pedicles were inserted with pedicle screws every level or every other level. The contralateral side pedicles were inserted with pedicle screws every two or three levels. Each fused vertebra had at least one pedicle screw. After confirmation of pedicle screws positions with portable X-ray device, rods were applied on the tulips of the screws. Partial facetectomy was performed at every level for local bone grafting. The major curve deformities were corrected by the derotation of the titanium rods, with hyperkyphotic contour rods were used at the concave side. Compression and distraction methods were used to assist correction of the proximal thoracic deformities and achieve shoulder balance. For deformities larger than 90°, cantilever bending was utilized for achieving better correction, especially in Lenke type 4 curves. The fixation constructs were all pedicle screw-based constructs. Crosslinks were routinely used at proximal and distal ends to reinforce the structure of the fixation construct. Posterior fusion was performed using local autologous bone chips and supplementary bone substitutes. The wounds were then closed in layers with the placement of a drainage tube.

Electroneurophysiological monitoring was performed during the operation. Standing and walking were encouraged on the second postoperative day with the application of thoracolumbar orthosis.

### Assessment of the results

The medical records of all patients were collected under the approval of the Institutional Review Board of our hospital. The curve patterns were classified according to the Lenke classification [19]. Preoperative and postoperative 2-year whole spinal column standing anteroposterior and lateral images were reviewed and analyzed to compare multiple radiographic parameters. For the coronal plane, major curve and structural curve Cobb angles were measured and recorded. Corresponding curve flexibilities were analyzed based on preoperative supine bending views of both sides. The curve correction rate and curve flexibility were defined as follows:

$$ \mathrm{Correction}\ \mathrm{rate}\ \left(\%\right)=\frac{\mathrm{Preoperative}\ \mathrm{Cobb}\ \mathrm{angle}-\mathrm{Postoperative}\ \mathrm{Cobb}\ \mathrm{angle}\ }{\mathrm{Preoperative}\ \mathrm{Cobb}\ \mathrm{angle}}\times 100\% $$

$$ \mathrm{Flexibility}\ \left(\%\right)=\frac{\mathrm{Preoperative}\ \mathrm{Cobb}\ \mathrm{angle}-\mathrm{Side}\ \mathrm{bending}\ \mathrm{Cobb}\ \mathrm{angle}}{\mathrm{Preoperative}\ \mathrm{Cobb}\ \mathrm{angle}}\times 100\% $$

To account for differences in curve flexibility, the Cincinnati curve correction index (CI) was analyzed, as described by Vora et al. [21]:

$$ \mathrm{Cincinnati}\ \mathrm{Correction}\ \mathrm{index}\ \left(\mathrm{CI}\right)=\frac{\mathrm{Curve}\ \mathrm{correction}\ \mathrm{rate}\ \left(\%\right)}{\mathrm{Curve}\ \mathrm{flexibility}\ \left(\%\right)} $$

Thoracic kyphosis (TK = T5-T12 Cobb angle) and lumbosacral lordosis (LL = L1-S1 Cobb angle) were measured in the sagittal plane. The axial rotation of major curve apical vertebra was measured using the Nash-Moe method [22]. The positions of major curve apical vertebral pedicles on standing whole spine anteroposterior radiographs were classified into the following 5 grades: 0, no asymmetry of the positions of pedicles; 1, the convex pedicle has medial migration and concave pedicle starts to disappear; 2, the convex pedicle continues medially migrating, and the concave pedicle has disappeared; 3, the convex pedicle touches the midline of the vertebral body; and 4, the convex pedicle migrates past the midline of the vertebral body. Coronal balance, sagittal balance, and apical vertebral translation were also calculated. Anchor density (AD) was defined as the number of total inserted pedicle screws divided by fusion levels.

### Statistical analysis

The correlation between the anchor density and the postoperative 2-year curve correction parameters in three dimensions (major curve correction rate, correction index, thoracic kyphosis correction, lumbar lordosis correction, apical vertebral rotation correction, and apical vertebral translation correction) were calculated using Pearson’s correlation coefficient. Subgroup analyses based on different curve types, curve magnitudes, and curve flexibilities were performed as followed: [1] All patients [2]; Main thoracic curves (MT, Lenke 1) [3]; Major MT curves ± other minor structural curves (Lenke 1–3) [4]; Thoracolumbar/lumbar curves (TL/L, Lenke 5) [5]; Major TL/L curves ± other minor structural curves (Lenke 5,6) [6]; Double structural MT and TL/L curves (Lenke 3,4,6) [7]; Small curve (major curve between 40° to 60°) [8]; Large curve (major curve over 60°) [9]; Stiff curve (flexibility ≤40%) [10]; Flexible curve (flexibility > 40%).

**Fig. 1:**
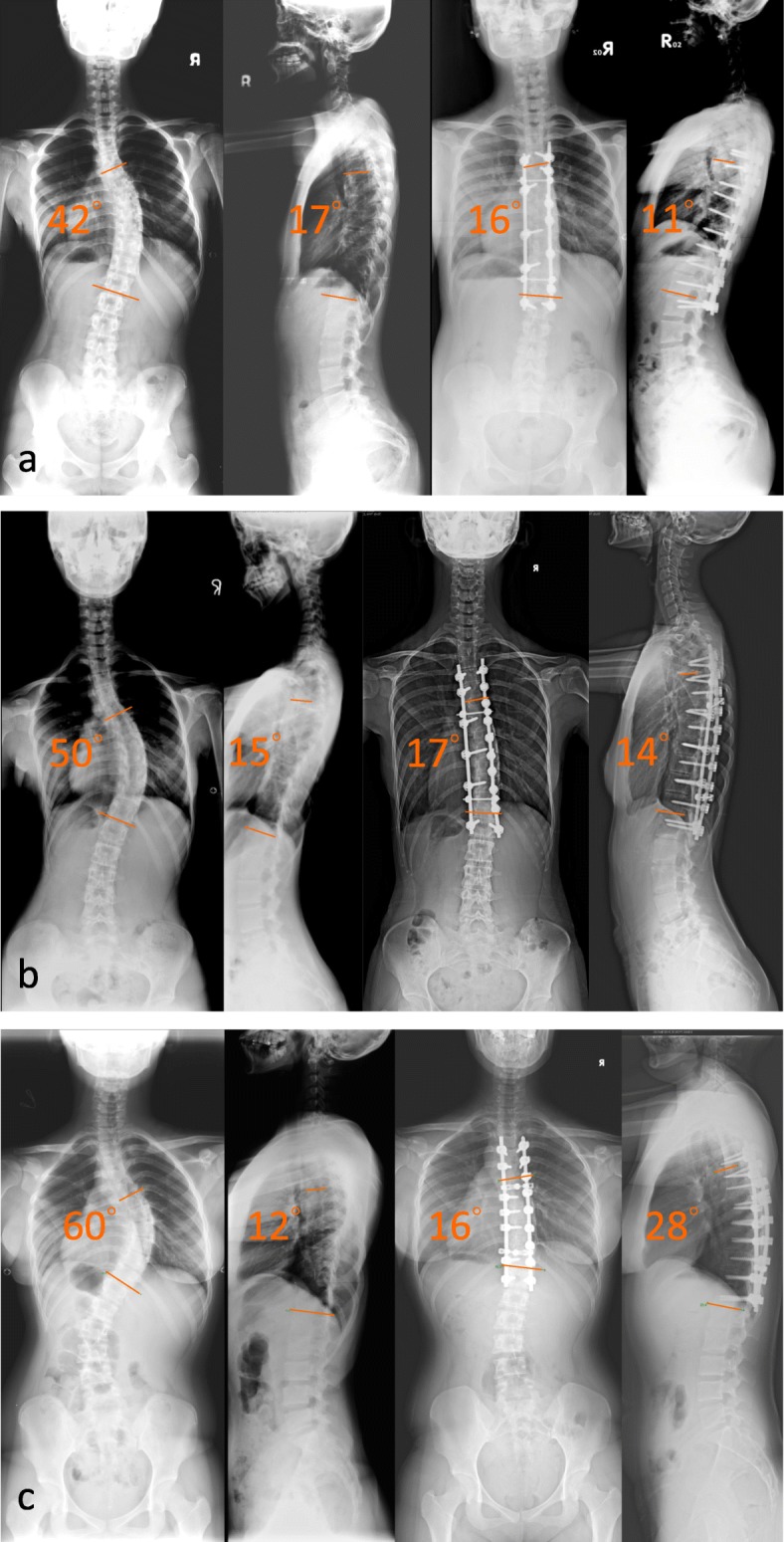
Low-density, Middle-density and High-density screw constructs. **a:** A patient with Lenke 1 AIS received posterior fusion of T4-L1 with a **low**-density construct (Anchor density = 1.20) of pedicle screw instrumentation. **b:** A patient with Lenke 1 AIS received posterior fusion of T4-L1 with a **middle**-density construct (Anchor density = 1.60) of pedicle screw instrumentation. **c:** A patient with Lenke 1 AIS received posterior fusion of T4-T12 with a **high**-density construct (Anchor density = 1.78) of pedicle screw instrumentation

To further investigate the impact of anchor density on curve correction, patients were divided into low-density (AD ≤1.4), middle-density (1.4 < AD ≤1.7) and high-density (AD > 1.7) groups (Fig. 1a, 1b & 1c) in different patient subgroups: [1] All patients [2]; Lenke 1–3 [3]; Lenke 1 patients. The statistical differences between low-density, middle-density, and high-density groups were tested using one-way analysis of variance (ANOVA) with Scheffé post-hoc tests. A *p* value of less than 0.05 was considered statistically significant in this study. All statistical tests were performed using SPSS v25 (IBM-SPSS, Armonk, NY).

## Results

A total of 127 patients (17 males and 110 females) aged 14.4 years old at the time of surgery were enrolled in the present study. The numbers of Lenke type 1, 2, 3, 4, 5 and 6 curve patients were 59, 19, 12, 6, 22 and 9, respectively. The mean anchor density of all patients was 1.60 (ranging from 1.14 to 2.0). The demographic data and radiographic parameters for all patients and Lenke 1–6 subgroup patients are shown in Table 1.

**Table 1:** All patients’ demographics and radiographic parameters

	All	Lenke 1	Lenke 2	Lenke 3	Lenke 4	Lenke 5	Lenke 6
Patients	127 (17M&110F)	59 (7M&52F)	19 (3M&16F)	12 (1M&11F)	6 (2M&4F)	22 (3M&19F)	9 (1M&8F)
Age	14.4 (10~24)	14.3 (10~22)	15.8 (11~24)	13.0 (11~15)	13.3 (12~15)	14.7 (11~23)	14.8 (11~22)
Level fused	9.9 ± 2.2	8.9 ± 1.1	12.2 ± 1.4	12.0 ± 2.1	13.3 ± 0.8	7.7 ± 1.1	11.9 ± 1.3
PreOP major curve	57.4° ± 13.3°	52.7° ± 8.2°	63.1° ± 10.9°	65.7° ± 14.6°	92.5° ± 13.0°	50.3° ± 6.2°	58.3° ± 10.9°
PostOP major curve	20.8° ± 8.6°	19.2° ± 5.6°	23.9° ± 7.7°	25.9° ± 5.7°	40.7° ± 15.6°	15.3° ± 6.2°	18.3° ± 5.3°
PreOP TK	20.8° ± 11.3°	17.8° ± 9.9°	20.4° ± 11.9°	25.2° ± 11.9°	29.5° ± 17.9°	24.4° ± 9.6°	20.7° ± 11.8°
PostOP TK	24.9° ± 10.2°	22.8° ± 8.1°	21.4° ± 9.4°	26.2° ± 9.7°	26.3° ± 17.4°	34.1° ± 9.3°	20.0° ± 9.1°
PreOP LL	54.0° ± 11.7°	53.7° ± 11.7°	52.3° ± 10.6°	55.4° ± 9.7°	53.5° ± 11.4°	54.9° ± 13.9°	55.2° ± 13.6°
PostOP LL	56.3° ± 12.2°	55.3° ± 11.3°	54.0° ± 11.9°	62.7° ± 12.7°	50.3° ± 6.0°	59.0° ± 14.9°	57.0° ± 11.2°
PreOP AVR	2.2 ± 0.6	1.9 ± 0.5	2.3 ± 0.6	2.0 ± 0.6	2.7 ± 0.8	2.7 ± 0.5	2.8 ± 0.7
PostOP AVR	1.4 ± 0.7	1.1 ± 0.5	1.6 ± 0.8	1.3 ± 0.5	2.3 ± 1.0	1.5 ± 0.7	1.4 ± 0.7
PreOP CB (mm)	-7.9 ± 17.0	-6.4 ± 14.6	2.8 ± 13.5	-15.3 ± 16.3	2.8 ± 12.4	-15.1 ± 21.5	-19.9 ± 13.5
PostOP CB (mm)	-8.8 ± 12.5	-9.3 ± 12.5	-1.6 ± 13.0	-11.6 ± 8.2	-6.7 ± 8.6	-11.1 ± 13.1	-12.3 ± 14.2
PreOP SB (mm)	-19.3 ± 32.9	-21.3 ± 32.8	-22.2 ± 35.5	-9.4 ± 27.9	-18.3 ± 48.6	-15.1 ± 33.5	-24.0 ± 25.6
PostOP SB (mm)	-14.6 ± 33.5	-16.0 ± 34.7	-14.9 ± 32.9	-11.5 ± 27.8	-11.6 ± 32.1	-12.0 ± 40.2	-17.3 ± 22.3
PreOP AVT (mm)	49.6 ± 13.8	45.1 ± 11.5	50.5 ± 13.4	46.8 ± 15.4	68.4 ± 19.5	57.0 ± 11.1	50.8 ± 13.3
PostOP AVT (mm)	23.2 ± 10.9	20.3 ± 9.1	23.9 ± 14.0	24.3 ± 10.2	40.1 ± 11.1	24.6 ± 10.0	24.1 ± 8.5
Anchor density^a^	1.60 (1.14~2)	1.59 (1.18~2)	1.48 (1.14~1.64)	1.61 (1.29~2)	1.67 (1.43~1.93)	1.74 (1.50~2)	1.59 (1.46~1.83)
Flexibility (%)	36.1 ± 19.3	36.1 ± 17.5	30.4 ± 13.1	18.0 ± 12.1	12.6 ± 8.7	51.9 ± 16.1	49.8 ± 20.8
Correction rate (%)	64.1 ± 10.1	63.6 ± 9.4	62.2 ± 9.3	59.9 ± 7.5	56.9 ± 11.1	69.8 ± 11.1	68.0 ± 10.0
Correction index	3.2 ± 5.2	3.0 ± 5.7	2.5 ± 1.1	6.5 ± 5.9	9.2 ± 10.7	1.6 ± 1.0	1.7 ± 1.1
TK correction	4.1° ± 9.7°	5.0° ± 9.9°	1.1° ± 7.0°	1.0° ± 7.9°	-3.2° ± 11.5°	9.8° ± 8.5°	-0.7° ± 11.3°
LL correction	2.4° ± 11.0°	1.6° ± 11.0°	1.7° ± 9.7°	7.3° ± 15.0°	-3.2° ± 12.3°	4.0° ± 10.6°	1.8° ± 6.6°
AVR correction	0.8 ± 0.6	0.7 ± 0.5	0.7 ± 0.5	0.8 ± 0.6	0.3 ± 0.8	1.2 ± 0.7	1.3 ± 0.7
AVT correction (mm)	26.5 ± 11.9	24.8 ± 10.8	26.6 ± 14.3	22.5 ± 12.8	28.4 ± 15.1	32.4 ± 10.7	26.6 ± 10.4

Data are presented as mean ± standard deviation (SD)

*M* male, *F* female, *OP* operative, *TK* thoracic kyphosis, *LL* lumbar lordosis, *AVR* apical vertebral rotation, *AVT* apical vertebral translation, *CB* coronal balance, *SB* sagittal balance

^a^Anchor density is presented as mean anchor density and its range

### Correlations between anchor density and curve correction in different curve types

According to the results shown in Table 2, anchor density was not correlated with coronal curve correction or apical vertebral rotation (AVR) correction (correction rate: r = − 0.01, *p* = 0.88; correction index: r = − 0.04, *p* = 0.63; AVR correction: r = 0.03, *p* = 0.75) in all patients. Nevertheless, no correlations existed between anchor density and the two-plane corrections in all of the subgroup analyses.

**Table 2:** Correlation coefficient between anchor density and all correction parameters

Parameters	All (N=127)		Lenke 1 (N=59)		Major MT (Lenke 1-3)(N=90)		Lenke 5 (N=22)		Major TL/L (Lenke 5,6)(N=31)		Double T&L (Lenke 3,4,6) (N=27)	
	r	*p*	r	*p*	r	*p*	r	*p*	r	*p*	r	*p*
Correction rate	-0.01	0.88	-0.04	0.76	-0.06	0.59	-0.35	0.11	-0.19	0.32	-0.30	0.12
Correction index	-0.04	0.63	-0.04	0.75	-0.01	0.97	-0.26	0.24	-0.07	0.71	-0.02	0.92
TK correction	0.27	0.002	0.31	0.02	0.27	0.01	0.40	0.06	0.33	0.07	0.12	0.56
LL correction	-0.07	0.45	-0.08	0.56	-0.11	0.32	0.16	0.47	0.06	0.74	-0.22	0.27
AVR correction	0.03	0.75	-0.24	0.07	-0.14	0.18	0.16	0.48	0.09	0.62	-0.14	0.47
AVT correction	0.01	0.90	-0.12	0.37	-0.15	0.15	0.43	0.03	0.31	0.10	-0.02	0.90

*Major MT* major main thoracic, *Major TL/L* major thoracolumbar/lumbar, *Double T&L* double thoracic and lumbar; *r* Pearson’s correlation coefficient; *p p* value

*TK* thoracic kyphosis, *LL* lumbar lordosis, *AVR* apical vertebral rotation, *AVT* apical vertebral translation

As for the sagittal curve correction, mild but positive correlations existed between anchor density and thoracic kyphosis correction in all patients (r = 0.27, *p* = 0.002), Lenke 1 patients (r = 0.31, *p* = 0.02) and patients with major MT curves ± other minor structural curves (Lenke 1–3; r = 0.27, *p* = 0.01). However, there were no correlations between anchor density and lumbar lordosis correction in Lenke 5 (r = 0.16, *p* = 0.47) and patients with major TL/L curves ± other minor structural curves (Lenke5–6; r = 0.06, *p* = 0.74).

In Lenke 5 patients, apical vertebral translation was positively correlated with anchor density (r = 0.43, *p* = 0.03). No correlations between apical vertebral translation and anchor density were found in the remaining subgroups.

For patients with double structural MT and TL/L curves (Lenke 3,4 & 6), there were no correlations between anchor density and curve corrections in all dimensions. (Table 2).

### Correlations between anchor density and curve correction in different curve magnitudes and flexibilities

No correlations existed between anchor density and coronal curve correction, correction index, lumbar lordosis (LL) correction, apical vertebral rotation (AVR) correction, or apical vertebral translation (AVT) correction in subgroup analysis of different curve magnitudes (small curves between 40°-60°, and large curves > 60°) and flexibilities (stiff curves with flexibility ≤40%, and flexible curves with flexibility > 40%). (Table 3).

**Table 3:** Correlations between anchor density and curve correction in different curve sizes and flexibilities

Parameters	Small curve (40°-60°)(N=89)		Large curve (>60°)(N=38)		Stiff curve (flexibility ≤ 40%) (N=78)		Flexible curve (flexibility > 40%)(N=49)	
	r	*p*	r	*p*	r	*p*	r	*p*
Correction rate	-0.04	0.69	-0.10	0.54	-0.04	0.73	-0.05	0.71
Correction index	-0.03	0.78	0.02	0.92	-0.04	0.71	-0.15	0.30
TK correction	0.38	<0.001	-0.06	0.72	0.24	0.06	0.34	0.01
LL correction	0.01	0.90	-0.15	0.39	-0.07	0.54	0.06	0.69
AVR correction	-0.04	0.70	-0.01	0.95	-0.08	0.48	0.07	0.66
AVT correction	0.05	0.65	0.01	0.96	-0.12	0.29	0.23	0.12

*r* Pearson’s correlation coefficient, *p p* value

*TK* thoracic kyphosis, *LL* lumbar lordosis, *AVR* apical vertebral rotation, *AVT* apical vertebral translation

Thoracic kyphosis (TK) correction was correlated with anchor density in small curves (r = 0.38, *p* <  0.001) and flexible curves (r = 0.34, *p* = 0.01). No correlations were observed between thoracic kyphosis (TK) correction and anchor density in large curves (r = − 0.06, *p* = 0.72) and stiff curves (r = 0.24, *p* = 0.06).

### Comparisons between different anchor density subgroups

The comparisons of fused level, flexibility and curve correction parameters between low-density, middle-density and high-density screw constructs are presented in Table 4. There were no differences between low-density, middle-density, and high-density in terms of coronal or axial curve correction parameters in all patients, Lenke 1–3 patients, and Lenke 1 patients. Significant differences between the three groups were observed in fused level and thoracic kyphosis correction.

**Table 4:** Different anchor density groups comparisons

	Low AD ≤ 1.4	Middle 1.4 < AD ≤ 1.7	High AD > 1.7	*p* value
All patients				
Patients	16 (2M&14F)	73 (7M&66F)	38 (8M&30F)	
Anchor density	1.31 ± 0.07	1.55 ± 0.08	1.83 ± 0.10	< 0.001
Fused level	10.6 ± 2.1	10.5 ± 2.1	8.4 ± 1.7	< 0.001
Flexibility (%)	32.0 ± 12.6	35.5 ± 20.8	38.9 ± 18.4	0.45
Correction rate (%)	64.3 ± 9.8	64.1 ± 9.5	63.9 ± 11.3	0.99
Correction index	2.5 ± 1.8	3.8 ± 6.5	2.4 ± 2.4	0.36
TK correction	-1.9° ± 8.8°	3.9° ± 9.7°	7.3° ± 9.1°	0.005
LL correction	2.4° ± 13.3°	2.5° ± 9.8°	2.1° ± 12.3°	0.99
AVR correction	0.7 ± 0.6	0.9 ± 0.6	0.8 ± 0.7	0.60
AVT correction (mm)	29.2 ± 13.2	25.7 ± 11.4	26.5 ± 11.9	0.57
Lenke 1-3				
Patients	16 (2M&14F)	54 (5M&49F)	20 (4M&16F)	
Anchor density	1.31 ± 0.07	1.54 ± 0.07	1.84 ± 0.10	< 0.001
Fused level	10.6 ± 2.1	10.4 ± 2.0	8.5 ± 0.9	< 0.001
Flexibility (%)	32.0 ± 12.6	33.2 ± 18.7	30.9 ± 16.0	0.88
Correction rate (%)	64.3 ± 9.8	62.4 ± 9.1	62.8 ± 9.1	0.77
Correction index	2.5 ± 1.8	3.7 ± 6.5	3.1 ± 3.1	0.73
TK correction	-1.9° ± 8.8°	4.1° ± 9.6°	6.9° ± 6.5°	0.01
LL correction	2.4° ± 13.3°	3.5° ± 10.6°	-0.5° ± 11.9°	0.42
AVR correction	0.7 ± 0.6	0.8 ± 0.5	0.6 ± 0.5	0.19
AVT correction (mm)	29.2 ± 13.2	24.7 ± 11.2	21.9 ± 11.6	0.18
Lenke 1				
Patients	10 (1M&9F)	31 (3M&28F)	18 (3M&15F)	
Anchor density	1.33 ± 0.06	1.53 ± 0.07	1.82 ± 0.09	< 0.001
Fused level	9.2 ± 0.9	9.1 ± 1.2	8.3 ± 0.8	0.03
Flexibility (%)	33.3 ± 13.6	39.1 ± 19.4	32.3 ± 15.5	0.37
Correction rate (%)	66.5 ± 9.2	62.6 ± 10.2	63.7 ± 8.1	0.53
Correction index	2.5 ± 1.8	3.4 ± 7.8	2.5 ± 1.4	0.85
TK correction	-3.6° ± 9.1°	6.6° ± 10.5°	7.0° ± 6.4°	0.008
LL correction	-2.3° ± 9.7°	4.8° ± 10.2°	-1.7° ± 11.9°	0.06
AVR correction	0.8 ± 0.6	0.8 ± 0.5	0.6 ± 0.5	0.24
AVT correction (mm)	27.8 ± 12.0	25.0 ± 10.4	22.9 ± 109	0.52

Data are presented as mean ± standard deviation (SD)

*AD* anchor density, *TK* thoracic kyphosis, *LL* lumbar lordosis, *AVR* apical vertebral rotation, *AVT* apical vertebral translation

Post-hoc Scheffé tests between different anchor density groups were shown in Table 5. In all patients, high-density group has significantly shorter fused level than low-density (mean difference 2.14, *p* = 0.001) and middle-density (mean difference 2.09, *p* <  0.001) groups, and larger thoracic kyphosis correction than low-density group (mean difference 9.25°, *p* = 0.004). Similar differences were observed in Lenke 1–3 patients, although low-density group has less thoracic kyphosis correction (mean difference 6.03°, *p* = 0.03) despite comparable fused level (mean difference 0.17, *p* = 0.94). Whereas in Lenke 1 patients, low-density has less thoracic kyphosis correction compared to middle-density (mean difference 10.21°, *p* = 0.01) and high-density (mean difference 10.60°, *p* = 0.01) groups without significant fused level differences.

**Table 5:** Post-hoc Scheffé tests between different anchor density groups

	Groups comparison	Mean difference	*p* value
All patients			
Fused level	Low > Middle	0.06	0.99
	Low > High	2.14	0.001
	Middle > High	2.09	< 0.001
TK correction	Low < Middle	5.64°	0.10
	Low < High	9.25°	0.004
	Middle < High	3.62°	0.14
Lenke 1-3			
Fused level	Low > Middle	0.17	0.94
	Low > High	2.11	0.003
	Middle > High	1.94	< 0.001
TK correction	Low < Middle	6.03°	0.049
	Low < High	8.79°	0.01
	Middle < High	2.76°	0.50
Lenke 1			
Fused level	Low > Middle	0.14	0.94
	Low > High	0.87	0.10
	Middle > High	0.73	0.06
TK correction	Low < Middle	10.21°	0.01
	Low < High	10.60°	0.01
	Middle < High	0.39°	0.99

Note: only the parameters with significant difference in analysis of variance were listed

*TK* thoracic kyphosis; Low: anchor density ≤ 1.4; Middle: 1.4 < anchor density ≤ 1.7; High: anchor density > 1.7

## Discussion

Pedicle screws instrumentation and posterior fusion have become the treatment of choice in AIS surgery [1–3]. Although, with the advancement of pedicle screw insertion techniques and electroneurophysiological monitoring systems, neurologic complications, increased intraoperative blood loss and implant costs remained a concern for spinal surgeons [4–6]. Correcting scoliotic deformity in the coronal plane is one of the earliest established surgical goals for AIS patients. The correlation of anchor density and AIS coronal curve correction has been widely studied and reported over the past decades. However, studies have shown contradictory results regarding the correlation of anchor density and coronal curve correction in AIS surgery [7–17].

Several studies have shown positive correlations between AIS coronal curve correction and anchor density. Clements et al. [7] reviewed 250 major thoracic and 42 major lumbar curves within all 6 Lenke types and observed weak but significant correlations with mixed types of implants. Yang et al. [9] proposed a similar weak correlation within 58 Lenke 1A and 1B patients. Chen et al. [11] further demonstrated a mild correlation (r = 0.43, *P* <  0.05) between thoracolumbar/lumbar curve correction and anchor density in 39 Lenke 5 AIS patients. A large series study that consisted of 584 Lenke 1, 245 Lenke 2 and 123 Lenke 5 AIS patients was conducted by Larson et al. [13] The results showed that the high anchor density (AD ≥1.54) group has a significantly better coronal curve correction than the low anchor density (AD < 1.54) group in Lenke 1 and Lenke 2 patients, and the significant difference continued to exist within postoperative 2 years. However, Lenke 5 patients did not demonstrate a significant difference in coronal curve correction between high- and low-density groups in the same study. Two studies in 2016 showed support for a positive correlation between coronal curve correction and anchor density. Ketenci et al. [15] studied 76 matched Lenke 1 patients and equally divided these patients into the consecutive pedicle screw group (mean anchor density 2) and the interval pedicle screw group (mean anchor density 1.14). A significantly better coronal and rotational correction of thoracic curve was observed in the consecutive pedicle screw group. Nevertheless, Mac-Thiong et al. [17] reviewed the coronal main curve and main thoracic curve correction of 137 AIS patients. The results showed that the curve correction of anchor density < 1.4 was significantly inferior to the curve correction of anchor density ≥ 1.8, whereas the curve correction of anchor density between 1.4 to 1.8 showed comparable results to an anchor density ≥ 1.8.

In contrast, several studies reported no correlations between coronal curve correction and anchor density. Quan et al. [8] reviewed 49 Lenke 1 patients and found no correlation between anchor density and coronal curve correction. Bharucha et al. [10] divided 91 Lenke 1 patients into 34 high-density and 57 low-density patients based on mean anchor density (1.3), and no differences of curve correction, thoracic kyphosis or apical thoracic rotation were observed. Gebhard et al. [12] also found no correlation between main thoracic curve correction and anchor density within 119 AIS patients. Rushton et al. [14] demonstrated no correlation between correction of coronal curve, thoracic kyphosis or lumbar lordosis in 106 AIS patients (78 Lenke 1). Kempaninem et al. [16] compared 26 high-density (mean anchor density 1.68) AIS patients with 26 low-density (mean anchor density 1.28) AIS patients and found no differences in major MT curve correction, coronal balance, sagittal balance or apical vertebral translation.

Our study results were more compatible with studies reporting no correlation between anchor density and coronal curve correction [8, 10, 12, 14, 16]. The coronal curve correction parameters in our study were not correlated with anchor density in all patients subgroups, with the account of different curve types, curve magnitudes and curve flexibilities. (Tables 2 & 3) Similarly, there were no differences between low-density, middle-density and high-density groups for coronal curves correction. (Table 4).

Spinal surgeons were more aware of the correction of thoracic kyphosis and lumbar lordosis since the introduction of three-dimensional correction concept of AIS surgery [1, 23]. Furthermore, hypokyphosis has been associated with pulmonary function compromise and risk of future proximal junctional kyphosis in AIS patients [24, 25]. Maintaining adequate lumbar lordosis is also essential to balanced sagittal profiles in long-segments instrumented spinal surgery [26]. Larson et al. [13] found high anchor density was associated with postoperative hypokyphosis in Lenke 1,2 AIS patients, while several other studies reported no correlations between anchor density and thoracic kyphosis/lumbar lordosis correction [14, 15, 17].

In our study, mild positive correlation existed between anchor density and thoracic kyphosis correction in all patients, Lenke 1 patients, and Lenke 1–3 patients. (Table 2) When divided into low-density, middle-density, and high-density groups, the differences in thoracic kyphosis correction was accompanied by fused level differences. (Table 4) Post-hoc Scheffé tests revealed that with about two addition fusion levels, the thoracic kyphosis correction was about 9° less in low-density group, as compared to high-density group. (Table 5) However, the thoracic kyphosis corrections were about 10° less with no significant differences in fusion level in low-density group when comparing to other two groups in Lenke 1 patients.

Several factors influence thoracic kyphosis corrections included preoperative thoracic kyphosis, main thoracic curve flexibility, anchor density of concave side, rod materials and facetectomy levels [27–29]. Although statistical radiographic differences of thoracic kyphosis correction were observed between low-density and other two groups, clinical significances of thoracic kyphosis correction were not reached since the mean differences were only 5°-10° between the three groups.

Studies have shown no significant correlations between anchor density and lumbar lordosis in instrumented thoracolumbar/lumbar curves [11, 13], which is compatible to our study in both Lenke 5 and patients with major TL/L curves ± other minor structural curves (Lenke 5–6). (Table 2) For patients with instrumentations in both structural thoracic and structural thoracolumbar/lumbar curves (Lenke 3,4,6), anchor density was not correlated with thoracic kyphosis (r = − 0.12, *p* = 0.55) and lumbar lordosis (r = − 0.22, *p* = 0.27) based on our study statistics.

The correction of the axial rotation of the deformed vertebrae is one of the key components of AIS surgery. Few studies have examined the relationship between anchor density and the rotational correction of axial plane. Bharucha et al. [10] reported no differences of trunk rotation correction between anchor density above or below 1.3 (mean anchor density of all 91 patients). Whereas Ketenci et al. [15] found consecutive pedicle screws construct (anchor density 2.0) has significant better apical vertebral rotation correction over interval pedicle screw construct (anchor density 1.14). In our study, there was no correlation between anchor density and apical vertebral rotation in all patients and all subgroups of different curve types, curve magnitudes and curve flexibilities. (Tables 2 & 3) Nevertheless, no differences were observed between low-density, middle-density, and high-density groups in terms of apical vertebral rotation correction (*p* = 0.60) in all patients. (Table 4).

To investigate the effect of anchor density in different curve magnitudes and flexibilities, Pearson correlation coefficients between anchor density and curve correction were calculated in small curves (40°-60°), large curves (> 60°), stiff curves (flexibility ≤40%) and flexible curves (flexibility > 40%) (Table 3). From the literatures [30, 31], the flexibility assessed by supine bending views were averaged more than 40%, which was set as the cutoff value between stiff curves and flexible curves in our study. In large and stiff curves, no correlations between anchor density and all curve correction parameters were observed. While thoracic kyphosis was positively correlated with anchor density in small curves (r = 0.38, *p* <  0.001) and flexible curves (r = 0.34, *p* = 0.01). Therefore, for small and flexible curves, increase the anchor density may result in more thoracic kyphosis correction. But the difference may not be obvious when the anchor density exceeds more than 1.7. However, for large and stiff curves, the anchor density was not correlated with all curve correction parameters, other correction techniques or longer fusion length were typically utilized to achieve adequate correction.

There were several limitations of this study, including the retrospective nature and lack of patient-reported outcome evaluations. The patients’ standing postures were not standardized in erect whole spine lateral view, which resulted in interpretation and reporting bias. Sagittal radiographic parameters were easily influenced by the patients’ positioning and motion [32, 33]. In addition, evaluating the axial vertebral rotation with the Nash-Moe method may not reflect the true axial rotation, since the classification only divides the rotations into four categories [22]. Further prospective studies and long-term surgical outcome comparisons can provide stronger evidence to clarify the true relationships between three-dimensional curve correction and anchor density.

## Conclusion

In our study, the anchor density was not related to coronal or axial curve corrections. Mild positive correlations with anchor density were found in thoracic kyphosis correction, especially in patients with smaller and flexible curves. Low anchor density with longer fusion level achieves similar curve corrections with middle or high anchor density in adolescent idiopathic scoliosis surgery. Therefore, spinal surgeons should consider the influences of anchor density on correcting deformities when planning the distributions of implants preoperatively.

## Data Availability

The datasets of this study are available from the corresponding author on reasonable request.

## Abbreviations

AD: Anchor density
AIS: Adolescent idiopathic scoliosis
AVR: Apical vertebral rotation
CI: Cincinnati curve correction index
LL: Lumbosacral lordosis
MT: Main thoracic
PT: Proximal thoracic
TK: Thoracic kyphosis
TL/L: Thoracolumbar/Lumbar
